# De Novo Transcriptome Assembly of *Agave* H11648 by Illumina Sequencing and Identification of Cellulose Synthase Genes in *Agave* Species

**DOI:** 10.3390/genes10020103

**Published:** 2019-01-30

**Authors:** Xing Huang, Mei Xiao, Jingen Xi, Chunping He, Jinlong Zheng, Helong Chen, Jianming Gao, Shiqing Zhang, Weihuai Wu, Yanqiong Liang, Li Xie, Kexian Yi

**Affiliations:** 1 Environment and Plant Protection Institute, Chinese Academy of Tropical Agricultural Sciences, Haikou 571101, China; hxalong@gmail.com (X.H.); xijingen@163.com (J.X.); hechunppp@163.com (C.H.); zhengjinlong_36@163.com (J.Z.); weihuaiwu2002@163.com (W.W.); yanqiongliang@126.com (Y.L.); 2 College of Plant Science and Technology, Huazhong Agricultural University, Wuhan 430070, China; xiaomei2016@163.com; 3 Institute of Tropical Bioscience and Biotechnology, Chinese Academy of Tropical Agricultural Sciences, Haikou 571101, China; chenhelong951@126.com (H.C.); gjmhust@163.com (J.G.); zsq04@126.com (S.Z.); 4 Institute of Tropical Agriculture and Forestry, Hainan University, Haikou, Hainan 570228, China; m13178981326@163.com

**Keywords:** RNA-seq, crassulacean acid metabolism, *Agave*, cellulose synthase, *Asparagus*, gene clone, phylogeny, gene expression

## Abstract

*Agave* plants are important crassulacean acid metabolism (CAM) plants with multiple agricultural uses, such as being used in tequila and fiber production. *Agave* hybrid H11648 ((*A*. *amaniensis* Trel. and Nowell × *A*. *angustifolia* Haw.) × *A*. *amaniensis*) is the main cultivated *Agave* species for fiber production in large tropical areas around the world. In this study, we conducted a transcriptome analysis of *A*. H11648. About 49.25 million clean reads were obtained by Illumina paired-end sequencing. De novo assembly produced 148,046 unigenes with more than 40% annotated in public databases, or matched homologs in model plants. More homologous gene pairs were found in *Asparagus* genome than in *Arabidopsis* or rice, which indicated a close evolutionary relationship between *Asparagus* and *A*. H11648. CAM-related gene families were also characterized as previously reported in *A. americana*. We further identified 12 cellulose synthase genes (*CesA*) in *Asparagus* genome and 38 *CesA* sequences from *A*. H11648, *A. americana*, *A. deserti* and *A. tequilana*. The full-length *CesA* genes were used as references for the cloning and assembly of their homologs in other *Agave* species. As a result, we obtained *CesA1*/*3*/*4*/*5*/*7* genes with full-length coding region in the four *Agave* species. Phylogenetic and expression analysis revealed a conserved evolutionary pattern, which could not explain the distinct fiber traits in different *Agave* species. We inferred that transcriptional regulation might be responsible for *Agave* fiber development. This study represents the transcriptome of *A*. H11648, which would expand the number of *Agave* genes and benefit relevant studies of *Agave* fiber development.

## 1. Introduction

*Agave* plants originated from the arid area of North America, with remarkable adaptation to the abiotic stresses of xeric environments [1]. As an important family of crassulacean acid metabolism (CAM) plants, the *Agave* genus contains many special biological characteristics that have been widely utilized in the production of alcoholic beverages, fiber, food, and medicinal compounds [2,3]. Its high water-use efficiency and multiple uses makes *Agave* an important agricultural and industrial crop that is suitable for sustainable production systems in warming and drying regions around the world [4,5]. Despite its huge potential, little is presently known about the physiology and molecular basis of the characteristics expressed in *Agave* plants. *Agave* species normally have very large genomes and are generally not fast-growing, with an extremely long life-cycle, which significantly challenges genetic improvement research efforts [6]. The development of next-generation sequencing technology has offered a great opportunity for researchers to reveal the *Agave* secrets [7]. A native plant to the Sonoran Desert, *A. deserti* has the ability to tolerate heat, drought, and cold, for which it has been characterized by for decades [8]. A series of candidate genes have been recently mined for adaptations to xeric environments by Illumina sequencing, which has potential value for the improvement of food crops [9]. In *A. americana*, the molecular timekeeping and evolution of CAM photosynthesis has been revealed by combined transcriptomic, proteomic and metabolomic approaches [10,11]. These data sets provide a resource, and the possibility of engineering CAM traits into main food crops. *A. tequilana* is mainly cultivated in Mexico to produce the spirit tequila and has been considered a cultural symbol for centuries [12]. Fructan is the main material in tequila production, and the main trait for genetic improvement in *A. tequilana*. Thus, several fructan-related gene families have been functionally characterized according to transcriptome mining [13,14]. Recently, transcriptome profiling was carried out, providing insight into the transcriptional dynamics of the plant leaf, root organs, and callus tissues, which improves our understanding of de novo organogenesis in *A. salmiana* [15]. There are only a few reports on studies that have been conducted on the molecular basis of fiber biosynthesis in *Agave* Hybrid H11648 ((*A. amaniensis* Trel. and Nowell × *A. angustifolia* Haw.) × *A. amaniensis*), a prominently cultivated cash crop in tropical regions around the world [6]. Therefore, RNA-seq is an efficient tool to obtain important genes that regulate fiber biosynthesis in *Agave*, such as cellulose synthase [16].

To provide a comprehensive and accurate description of transcripts, we conducted a transcriptome analysis of leaf samples in *A*. H11648 by Illumina paired-end sequencing. Cellulose synthase (*CesA*) genes were further identified in *A*. H11648, *A. americana*, *A. deserti* and *A. tequilana*. Phylogenetic and expression analysis were also carried out to estimate the evolutionary pattern of *Agave CesA* genes. From this, our results aimed to expand the number of *Agave* genes and benefit relevant studies of *Agave* fiber development.

## 2. Materials and Methods

### 2.1. Plant Materials and RNA Extraction

The plants of *A*. H11648, *A. americana* Marginata, *A. deserti* and *A. tequilana* were grown and normally managed in the Wenchang experimental field (19°32′19″ N 110°46′08″ E) of Environment and Plant Protection Institute, Chinese Academy of Tropical Agricultural Sciences, since 2013. Immature leaf samples (leaf length within 20-cm, distal part) were collected from the top of 3-year-old plants. Three plants of each *Agave* species were sampled as biological replicates. The sampled leaves were immediately placed into liquid nitrogen and stored at −80 °C. Total RNAs were extracted from each leaf sample using a Tiangen RNA prep Pure Plant Kit (Tiangen Biomart, Beijing, China) according to the manufacturers protocol.

### 2.2. Library Construction and Illumina Sequencing

Three RNA samples of *A*. H11648 leaf were mixed together with equal volumes and were sent to Genoseq Technology Co. Ltd. (Wuhan, Hubei, China) for library construction and Illumina paired-end sequencing. A total of 10 μg qualified RNA was used for the transcriptome library, as was previously reported [17]. The total RNAs extracted from the samples were purified by poly-T oligo-attached magnetic beads to obtain mRNAs, which were subsequently fragmented by TruSeq RNA Sample Prep Kit (Illumina, San Diego, CA, USA). First strand cDNA was synthesized by random hexamer primer and M-MuLV Reverse Transcriptase (RNase H). DNA Polymerase I and RNase H were used for second-strand cDNA synthesis. Single ‘A’ bases and ligation of adaptors were added to these double-stranded cDNA fragments. The fragments with adaptors were selected by gel purification and utilized for PCR amplification to generate a cDNA library. Transcriptome sequencing was conducted by Illumina HiSeq platform, with 150 bp paired–end raw reads.

### 2.3. De Novo Assembly and Functional Annotation

The raw reads under the accession number SRP132128 were deposited to the Sequence Read Archive [18]. We used Cutadapt for removing adaptor sequences and Trimmomatic for filtering low quality sequences with default settings [19,20]. The remaining clean reads were utilized for de novo transcriptome assembly by Trinity [21]. The non-redundant sequences of transcriptome were annotated in public databases, such as the Gene Ontology (GO), the Kyoto Encyclopedia of Genes and Genomes (KEGG), the euKaryotic Ortholog Groups database (COG ), the NCBI non-redundant protein database (Nr) and the Universal Protein Resource (UniProt), by Blastx method, with a cut-off Expect value (E value) of 10^−5^ [22,23,24,25,26]. These sequences were further utilized for homologous gene comparison with genome databases of *Arabidopsis thaliana*, *Oryza sativa,* and *Asparagus officinalis* in NCBI by Blastx method, with a cut-off E value of 10^−5^ [22].

### 2.4. Identification and Cloning of CesA Genes

*Arabidopsis* and rice *CesA* proteins were obtained according to the methods described in previous studies (Table S1) [27,28]. They were used to search against *Asparagus* genome for *CesA* genes by using the TBlastn method [22]. *Arabidopsis*, rice and *Asparagus CesA* proteins were utilized as queries to search against the transcriptomes of *A*. H11648, *A. deserti, A. tequilana* [9], and *A. americana* [10], respectively. Any *Agave* homologous sequences with full-length coding regions were listed and used as references for the reassembly of *CesA* genes in other *Agave* species. Multiple sequences of each of the *CesA* genes from different *Agave* species were reassembled, as previously reported [29]. Sequences with overlap of more than 20 bp were considered as partial sequences of the same gene. Gaps were further cloned according to full-length homologous genes in other *Agave* species.

Primers (Table S2) for gap sequence cloning were designed according to flanking sequence of the gaps or conserved regions of homologous genes in other *Agave* species by Oligo software version 7.56 [30]. The RNA samples of each *Agave* species were used for reverse cDNA synthesis with a GoScript Reverse Transcription System (Promega, Madison, WI, USA), according to the manufacturers instruction. Each reaction contained 1 μL of cDNA template, 1 μL of primers (10 μM), 5 μL EasyTaq Buffer (Transgen Biotech, Beijing, China), 4 μL dNTPs (2.5 mM), 0.5 μL EasyTaq DNA Polymerase (Transgen Biotech, Beijing, China), and 7.5 μL of ddH_2_O, with a final volume of 20 μL. The PCR procedure was: 94 °C for 5 min, followed by 30 cycles of 94 °C for 30 s, 60 °C for 30 s, 72 °C for 30 s, and 72 °C for 5 min after all cycles. The amplified products of gap sequences in 7 *Agave CesA* genes were sequenced for reassembly (Table S3). The reassembled *Agave CesA* genes were named according to their homologs in *Arabidopsis* and rice, and then deposited to Genbank [31]. The details of these genes have been listed in Table S3.

### 2.5. Phylogenetic Analysis

All *CesA* protein sequences from *Arabidopsis*, rice, *Asparagus* and *Agave* species were utilized for phylogenetic analysis. The Maximum Likelihood (ML) method was selected to construct phylogenetic tree with MEGA 5.0 software [32]. Bootstrap values from 1000 trails were used for the most parsimonious tree. These protein sequences were further aligned to investigate conserved motifs by using DNAMAN 7 software [33].

### 2.6. Expression Analysis

For transcriptome expression in *A*. H11648, RSEM was used to calculate the count of clean reads mapped to unigenes [34]. All read counts were normalized to reads per kilo-bases per million mapped reads (RPKM) [35]. The RPKM values of *A. deserti*, *A. tequilana*, and *A. americana* were obtained from previous studies [9,10]. RT-qPCR validation was conducted by a QuantStudio 6 Flex Real-Time PCR System (Thermo Fisher Scientific, Waltham, MA, USA). Each reaction contained 1 μL of cDNA template, 0.5 μL of gene-specific primers (10 μM), 10 μL TransStart Tip Green qPCR Supermix (Transgen Biotech, Beijing, China), 0.4 μL Passive Reference Dye (50×) (Transgen Biotech, Beijing, China), and 7.6 μL of ddH_2_O, with a final volume of 20 μL. The *protein phosphatase 2A* (PP2A) gene was selected as the endogenous control [36]. Gene-specific primers were designed for each *CesA* genes according to the conserved regions in *Agave* species by Oligo software version 7.56 [30]. The thermal cycle was as follows: 94 °C for 30 s, followed by 40 cycles of 94 °C for 5 s and 60 °C for 30 s. A dissociation stage was carried out after 40 cycles. Each sample had three replicates. Relative expression levels were calculated according to a method described in a previous study [37].

## 3. Results

### 3.1. Illumina Sequencing and De Novo Assembly

Leaves are the main vegetative part above ground and are used for fiber production in *A*. H11648. Thus, total RNA was extracted from the leaf for Illumina paired-end sequencing. As a result, a total of 60,791,648 raw reads were generated. After data filtration, 49.25 million clean reads were obtained, with 48.84% GC content and 96.10% Q30 bases (base quality >30). De novo assembly produced 148,046 unigenes with a N50 length at 591 bp and a total length at 76.78 Mb (Table S4). Among these, there were 133, 281, 10,026, and 4739 unigenes, with lengths ranging from 200–999 bp, 1000–2000 bp, and >2000 bp, respectively (Figure S1).

### 3.2. Functional Annotation

**Figure 1:**
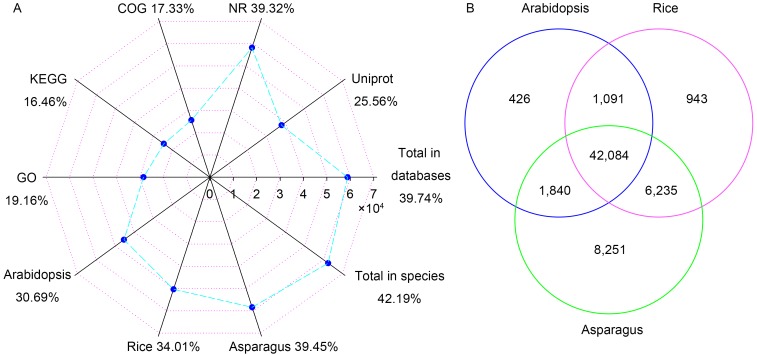
Numbers and rates of unigenes that were annotated in 5 public databases and 3 plant species (**A**). Numbers of unigenes that matched homologs in *Arabidopsis*, rice and *Asparagus* (**B**).

**Figure 2:**
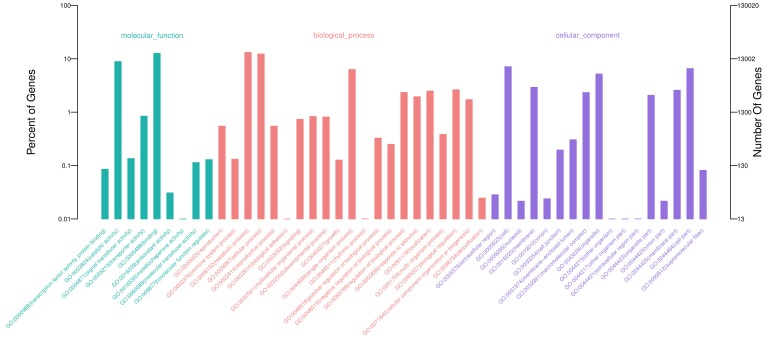
Gene Ontology (GO) classifications for the assembled unigenes.

All the unigenes were compared to public databases and published plant genomes, and the results have been listed in Table S4. In this study, 58,839 unigenes were annotated in at least one public database and 62,457 unigenes matched homologous genes in at least one of the three plant genomes (Figure 1A). There were 58,206 (39.32%) and 37,847 (25.56%) unigenes that demonstrated a similarity to known genes in Nr and Uniprot databases, respectively. Based on GO, 28,372 (19.16%) unigenes were categorized into 3 main categories and 47 subcategories (Figure 2). The eight most abundant subcategories were metabolic process (17,137), binding (16,485), cellular process (15,995), catalytic activity (11,543), cell (9242), cell part (8537), single-organism process (8182) and organelle (6749). From KEGG, 24,366 unigenes were assigned into 31 pathways (Figure S2). The most represented pathways were translation (2647), folding, sorting and degradation (2175), energy metabolism (1955), and carbohydrate metabolism (1915). In COG, 25,650 unigenes were clustered into 26 classifications (Figure S3). The classifications of general function prediction only (10,046), posttranslational modification, protein turnover, chaperones (3296), and signal transduction mechanisms (2913) were the most abundant. Among the 63,457 unigenes from genome searching, 42,084 unigenes had homologs in all three genomes (Figure 1B). There were 1091 homologous pairs between *Arabidopsis* and rice, 1840 between *Arabidopsis* and *Asparagus*, and 6235 between rice and *Asparagus*. Besides that, 426, 943, and 8251 homologous pairs were found in *Arabidopsis*, rice, and *Asparagus*, respectively.

### 3.3. Identification and Cloning of CesA Genes

**Figure 3:**
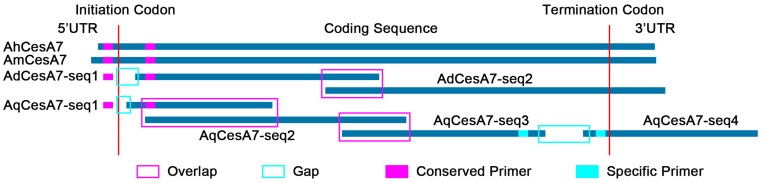
A sketch map for the cloning of cellulose synthase genes *(CesA)* in *Agave* plants (*CesA7* as an example).

*CesA* proteins from model plants (*Arabidopsis* and rice) were selected as queries to search the *Asparagus* genome. After removing redundant sequences, 12 *CesA* genes were found in the *Asparagus* genome and named according to their chromosome locations (Table S1). As a closely related Asparagaceae plant, *Asparagus CesA* proteins were also used to search *Agave* genes, together with *Arabidopsis* and rice. Eight full-length genes of *CesA1*/*3*/*4*/*5*/*7* were found in at least one of the four *Agave* species and 30 partial sequences (Table S3). These full-length genes were used as references for the cloning and assembly of their homologs in other *Agave* species (Figure 3). Two gaps were found in *AqCesA7* and one gap was found in *AqCesA1*/*3*/*5*, *AmCesA3*, and *AdCesA7* (Table S3). These gap sequences were further amplified and sequenced by the Sanger method. After the removal of overlap and gap filling, we obtained *CesA1*/*3*/*4*/*5*/*7* genes with full length in each *Agave* species.

### 3.4. Phylogeny of CesA Genes

**Figure 4:**
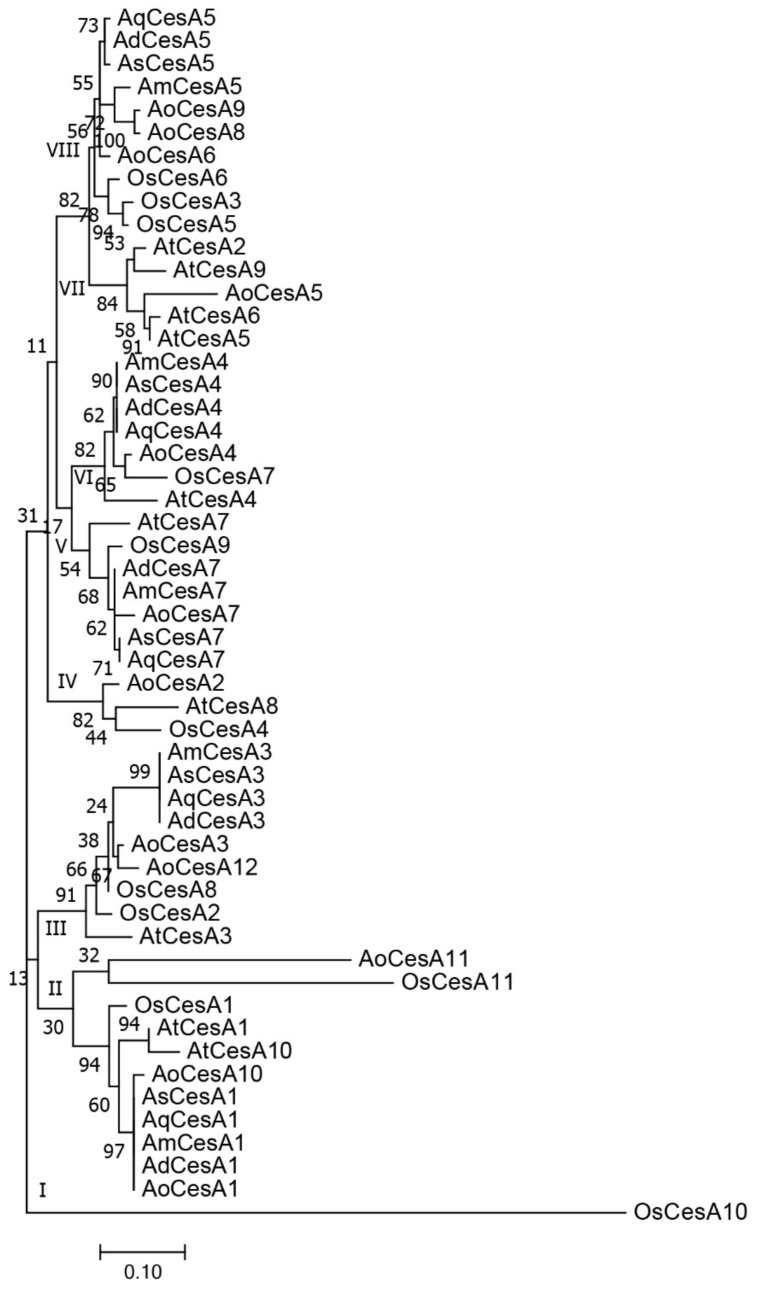
Phylogenetic analysis of *CesA* proteins in *Arabidopsis* (**At**), rice (**Os**), *Asparagus* (**Ao**), *A*. H11648 (**Ah**), *A. deserti* (**Ad**), *A. americana* (**Am**), and *A. tequilana* (**Aq**).

All the *CesA* proteins of the seven plants were utilized in phylogenetic analysis. All the 53 sequences were clustered into eight groups (Figure 4). *Agave* sequences were grouped together in each subgroup, which was commonly accompanied with an *Asparagus* sequence. There were also several subgroups without *Agave* sequences, such as groups I, IV, and VII. These protein sequences were further aligned to search conserved domains, and 45 conserved amino acid residues were found (Figure S4). We further compared the chromosome location of *CesA* genes in *Arabidopsis*, rice, and *Asparagus*. Our results indicated *CesA* genes had stable amounts and tended to disperse with increased chromosome numbers in different plants (Figure S5).

### 3.5. Expression Pattern of Agave CesA Genes

**Figure 5:**
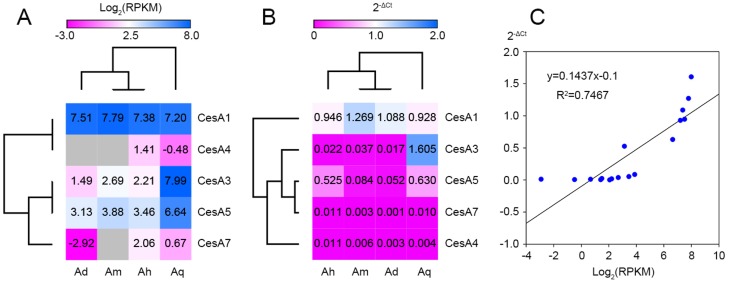
Expression analysis of *Agave CesA* genes by RNA-seq (**A**), RT-qPCR (**B**) and correlation analysis between them (**C**). Blanked squares represent no data.

According to transcriptome expression analysis, *CesA1* genes were very similarly expressed in each *Agave* species (Figure 5A). *CesA3*/*5* showed a higher expression level in *A. tequilana* than the other *Agave* species, as well as *CesA4*/*7* in A. H11648. For RT-qPCR validation, four *CesA1* genes and *AqCesA3* showed similar expression patterns with RNA-Seq results (Figure 5B). Besides, *CesA5* showed higher expression levels in *A*. H11648/*A. tequilana* than in *A. deserti*/*A. americana*. We further carried out a correlation analysis between RNA-seq and RT-qPCR results. The correlation coefficient was 0.7467 (R^2^), which was significant at the 0.01 level (Figure 5C).

## 4. Discussion

### 4.1. Characterization of the A. H11648 Transcriptome

With the development of sequencing technology, more plants have been sequenced to obtain the availability of genome data, such as the newly released genomes of *Asparagus* [38], sunflower [39], tea [40], etc. Recently, the project concerning 10,000 plant genomes and nanopore sequencing technology has significantly accelerated plant genome research [41,42]. However, *Agave* genomes are still too large to be assembled, which makes transcriptome an efficient tool for gene mining and marker development [6,43]. To date, there are only a few EST sequences of *A*. H11648 deposited into NCBI, which has restricted our knowledge of *Agave* fiber development. In our study, we successfully obtained the transcriptome and discovered 148,046 unigenes, which has significantly enlarged the bio-information of *A*. H11648. The unigene amount is similar to *A. deserti* and *A. tequilana* [9], but higher than *A. americana* [10]. This might have been caused by different samples and methods for transcriptome assembly [44]. Our data size was relatively lower than the 3 species, which would have affected assembly quality [45]. However, there are still 14,765 unigenes with a sequence length over 1000 bp. These long sequences deserve to be focused on in future studies, and we found several full-length, or partial, *CesA* genes from analyzing them (Table S3).

In addition, about 40% of all the unigenes were annotated in public databases (Figure 1A). These would help us to mine candidate genes for important agronomic traits in *A*. H11648 and construct their regulation networks, especially the mechanism of leaf fiber formation and development. CAM-related genes associated with circadian rhythm plant (114), citrate cycle (230), pyruvate metabolism (309), and photosynthesis (225) (Table S4) were characterized in KEGG results based on previous studies conducted on *A. americana* [10,11]. These genes provide new proof for the conserved CAM evolution in *Agave* plants. Interestingly, there are more unigenes with homologs in the species than have been annotated in public databases, which implies that there are still many unknown genes not yet deposited to public databases. Homologs of *A*. H11648 that matched *Arabidopsis*, rice, and *Asparagus* were counted, and these numbers successively increased in the three plants (Figure 1B). This indicates a closer relationship between *A*. H11648 and *Asparagus* than to *Arabidopsis*/rice. Both of the subfamilies Agavoideae and Asparagoideae belong to the family Asparagaceae. [46]. The availability of the *Asparagus* genome will be an important reference for gene mining and genome assembling of *Agave* plants [38].

### 4.2. Potential Candidate CesA Genes Involved in Leaf Fiber Biosynthesis

Cellulose is one of the main components in the plant cell wall. Cellulose synthase plays a leading role in cellulose biosynthesis, including cellulose initiation and elongation [47]. The cellulose synthase catalytic sub-units encoded by *CesAs* are the core catalysts which generate the cellulose of the plant cell wall [16]. Thus, *CesA* genes have been studied in many plants, such as model plants *Arabidopsis* [27], and rice [28], especially in fiber crop cotton [48] and ramie [49]. Until now, only a few *CesA* genes have been reported in *A*. H11648, despite its importance as a fiber-producing plant in tropical areas. It has been reported that *A. deserti* and *A. tequilana* genes showed a significant similarity [9]. For this reason, we selected the three published *Agave* transcriptomes for screening *CesA* genes. We did find full-length *CesA* genes in at least one *Agave* species, as expected. Partial sequences were further cloned or reassembled, with the full-length genes as reference. As a result, we successfully cloned 20 full-length *CesA* genes in the four *Agave* species, which was far more efficient than the identification of partial *CesA* genes in ramie [49]. This result indicates that it is particularly important to clone genes with a closely related species as reference.

Moreover, we conducted evolutionary and expression analysis to evaluate the difference of *CesA* genes. The phylogenetic analysis and sequence alignment have revealed a conserved evolutionary pattern of *CesA* genes in different plants, as reported in previous studies [50]. Our results also indicated the close relationship between *Asparagus* and *Agave* plants. According to the syntenic chromosomal comparison between the three model plants (Figure S5), we inferred that there might be at least ten *CesA* genes in *Agave* genomes. According to expression analysis, most *CesA* genes were expressed at similar levels in leaves of different *Agave* species, except for *AqCesA3* (Figure 5). However, this proof is unable to explain the distinct fiber traits in *Agave* species, which means there must be complex transcription regulations during fiber development [51]. More samples of different tissues and developmental stages are still needed for further characterization of their expression patterns. These *Agave CesA* genes have brought about a great opportunity to understand the mechanisms of leaf fiber formation and development.

## 5. Conclusions

In our study, we successfully conducted a de novo transcriptome assembly of *Agave* H11648 by Illumina sequencing. A total of 148,046 unigenes were characterized, providing significant bioinformation and a strong foundation for future genomic studies in *A*. H11648. Furthermore, 38 *CesA* sequences were identified. Among these sequences, the full-length *CesA1*/*3*/*4*/*5*/*7* genes were cloned in *A*. H11648, *A. americana*, *A. deserti*, and *A. tequilana*, respectively. Phylogenetic and expression analysis revealed a conserved evolutionary pattern, which could not explain the distinct fiber traits in different *Agave* species. We therefore inferred that transcriptional regulation should be responsible for *Agave* fiber development. The identification of *CesA* genes has improved our understanding and remains relevant for future studies on the formation and development of *Agave* leaf fiber.

## Supplementary Materials

The following are available online at http://www.mdpi.com/2073-4425/10/2/103/s1, Figure S1: Length distribution of the assembled unigenes. Figure S2: KEGG classifications for the assembled unigenes. Figure S3: COG classifications for the assembled unigenes. Figure S4: Alignment of *CesA* proteins in the 7 plants. Conserved amino acid residues are highlighted in black color. Figure S5: Comparative maps of *CesA* genes and their homologous genes within syntenic chromosomal intervals between *Arabidosis* (*blue*), rice (*pink*), and *Asparagus* (*green*). The numbers within or beside colored bars represents chromosome numbers and *CesA* gene numbers, respectively. Conserved gene pairs are connected with dotted lines. Table S1: Details of *CesA* genes in *Arabidopsis*, rice and *Asparagus*. Table S2: Primers used for cloning and RT-qPCR of *Agave CesA* genes. Table S3: Details and reassembly of *Agave CesA* genes. Table S4: Details and annotation information (in GO, KEGG, COG, Nr and Uniprot) and homolog information (in *Arabidopsis*, rice and *Asparagus*) of unigenes.
