# Supplementary figures and images for: De Novo Transcriptome Assembly of Agave H11648 by Illumina Sequencing and Identification of Cellulose Synthase Genes in Agave Species
Source: Genes (Basel). 2019 Jan 30;10(2):103. doi: 10.3390/genes10020103 (PMC6409920; doi:10.3390/genes10020103)

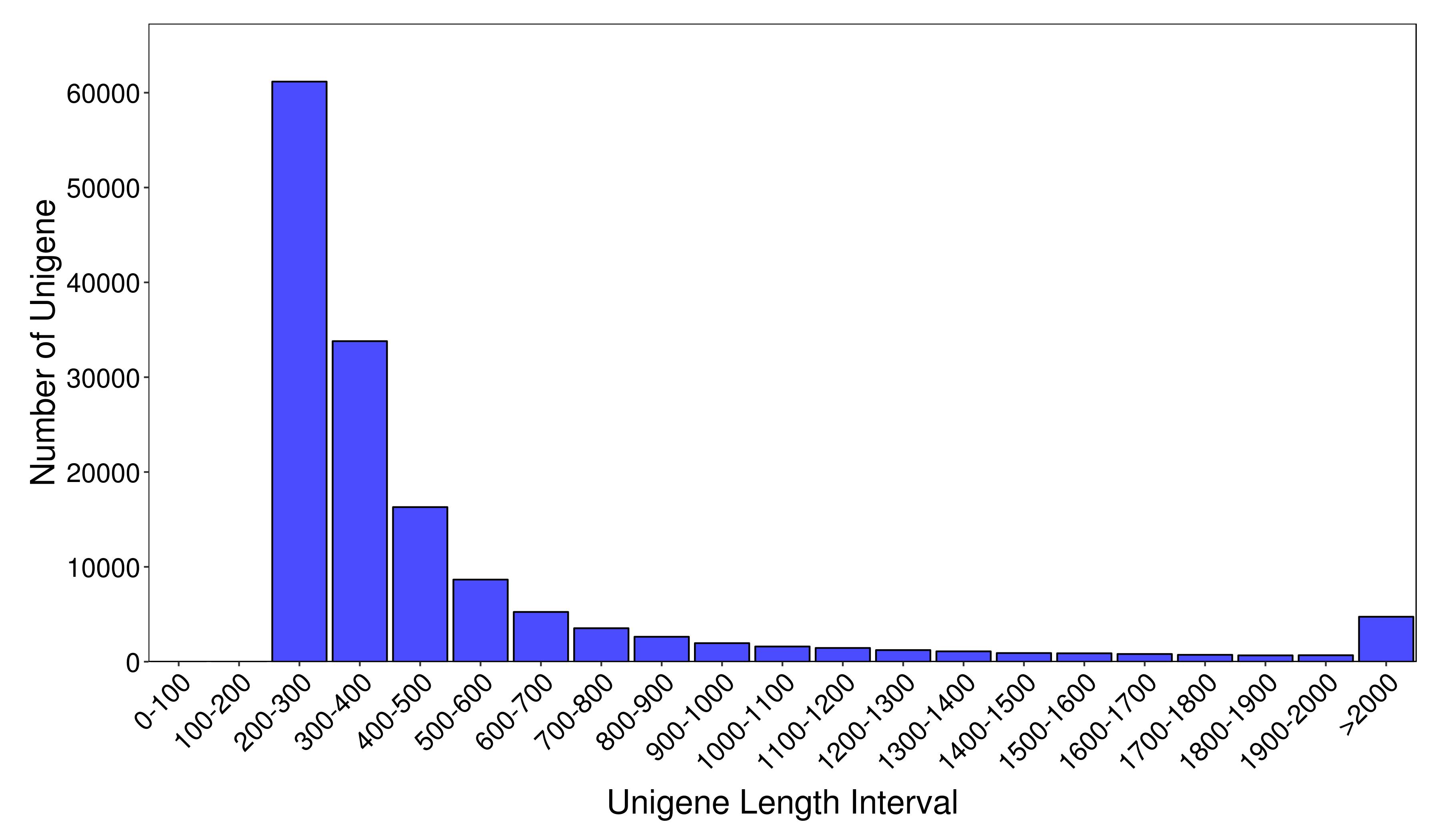

Supplement: Supplementary file 1 [file genes-10-00103-s001.zip › Figure S1.jpg]

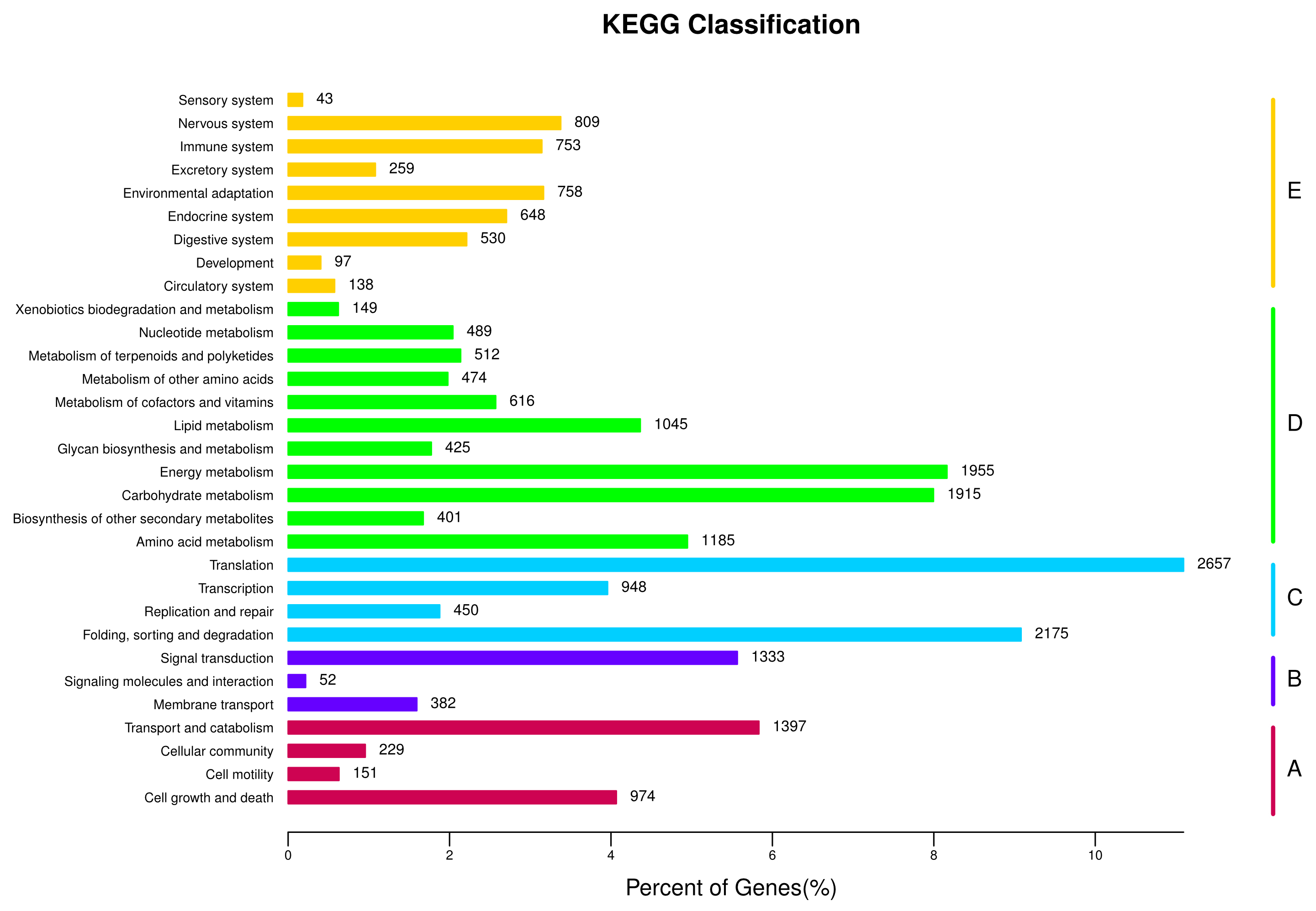

Supplement: Supplementary file 1 [file genes-10-00103-s001.zip › Figure S2.jpg]

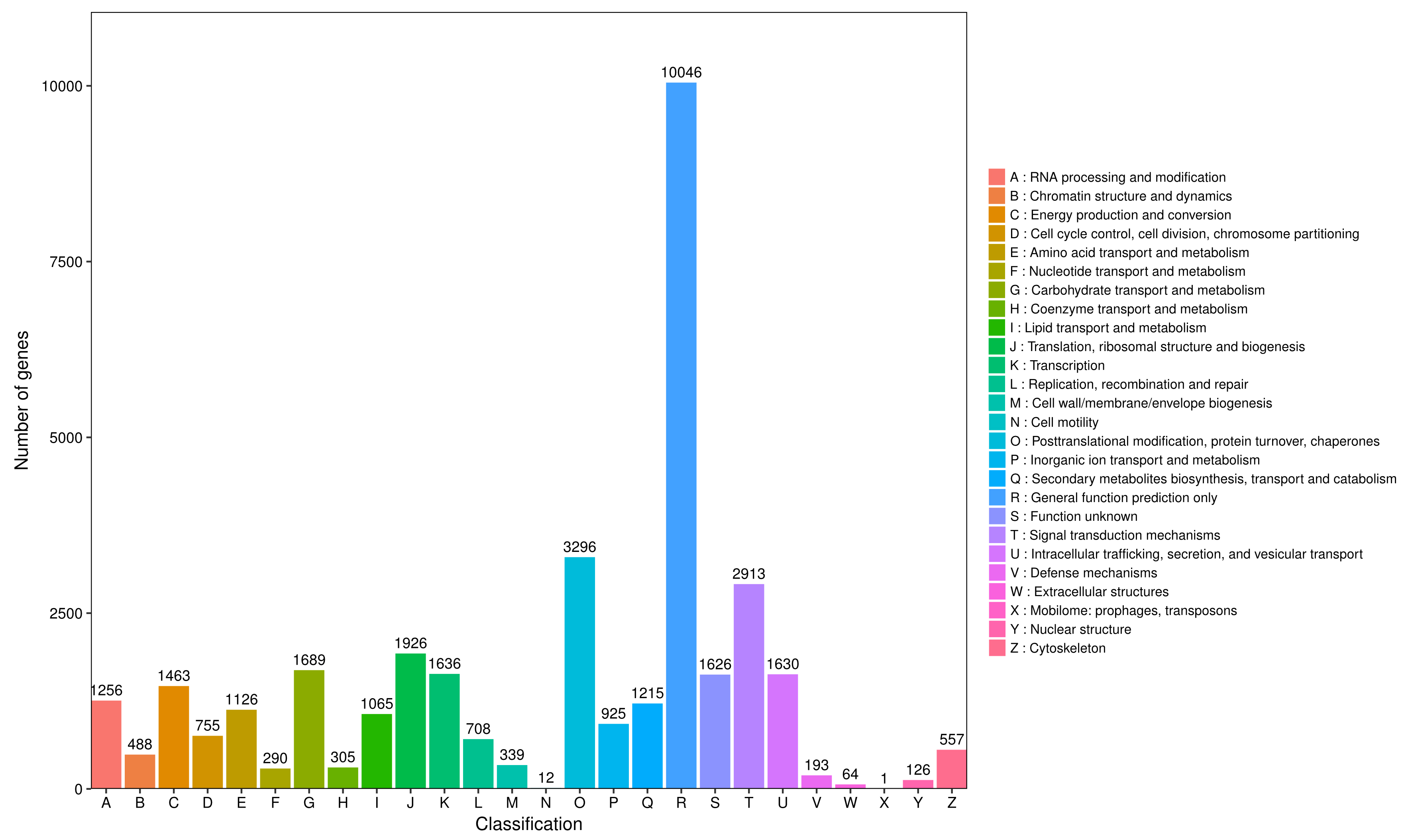

Supplement: Supplementary file 1 [file genes-10-00103-s001.zip › Figure S3.jpg]

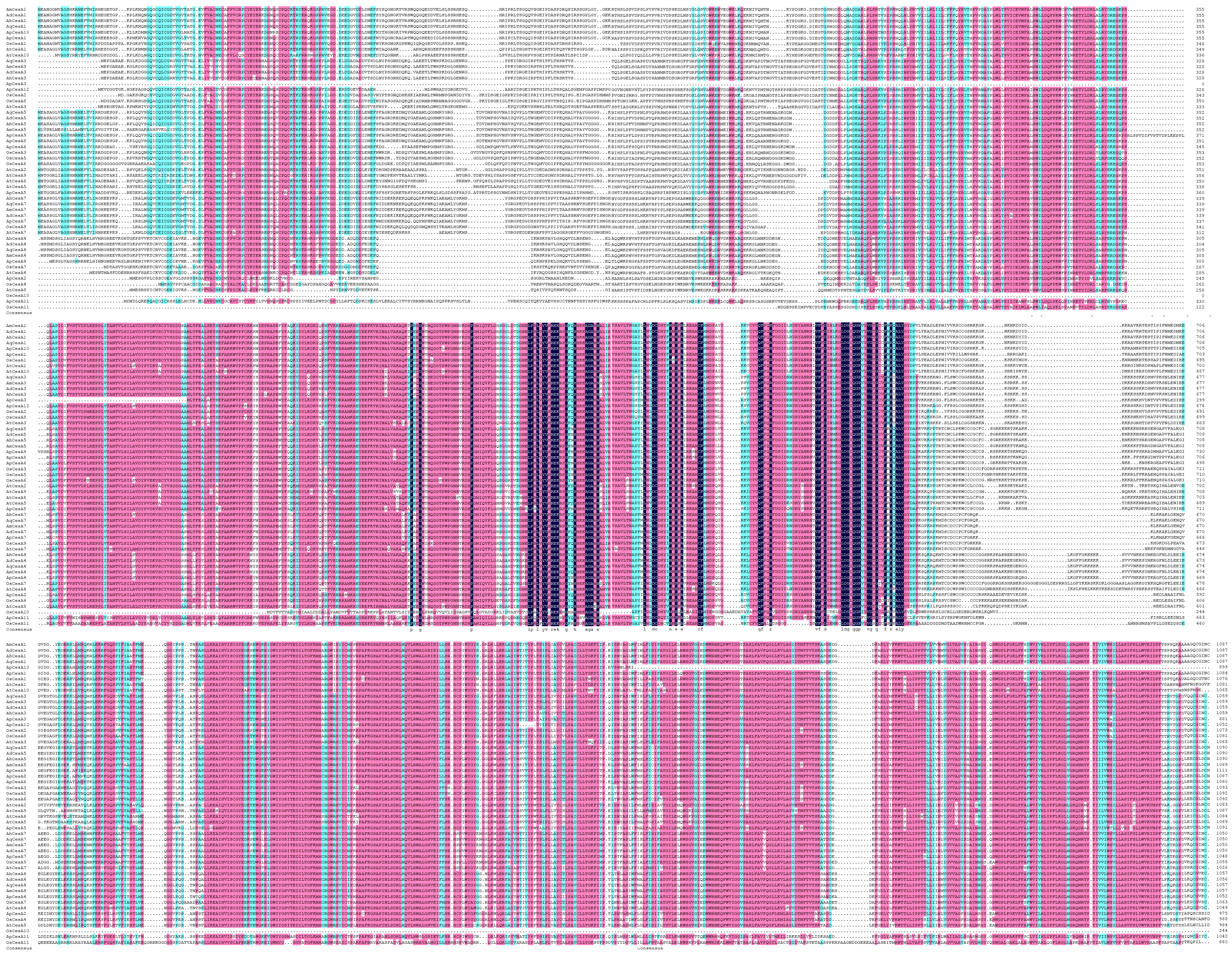

Supplement: Supplementary file 1 [file genes-10-00103-s001.zip › Figure S4.jpg]

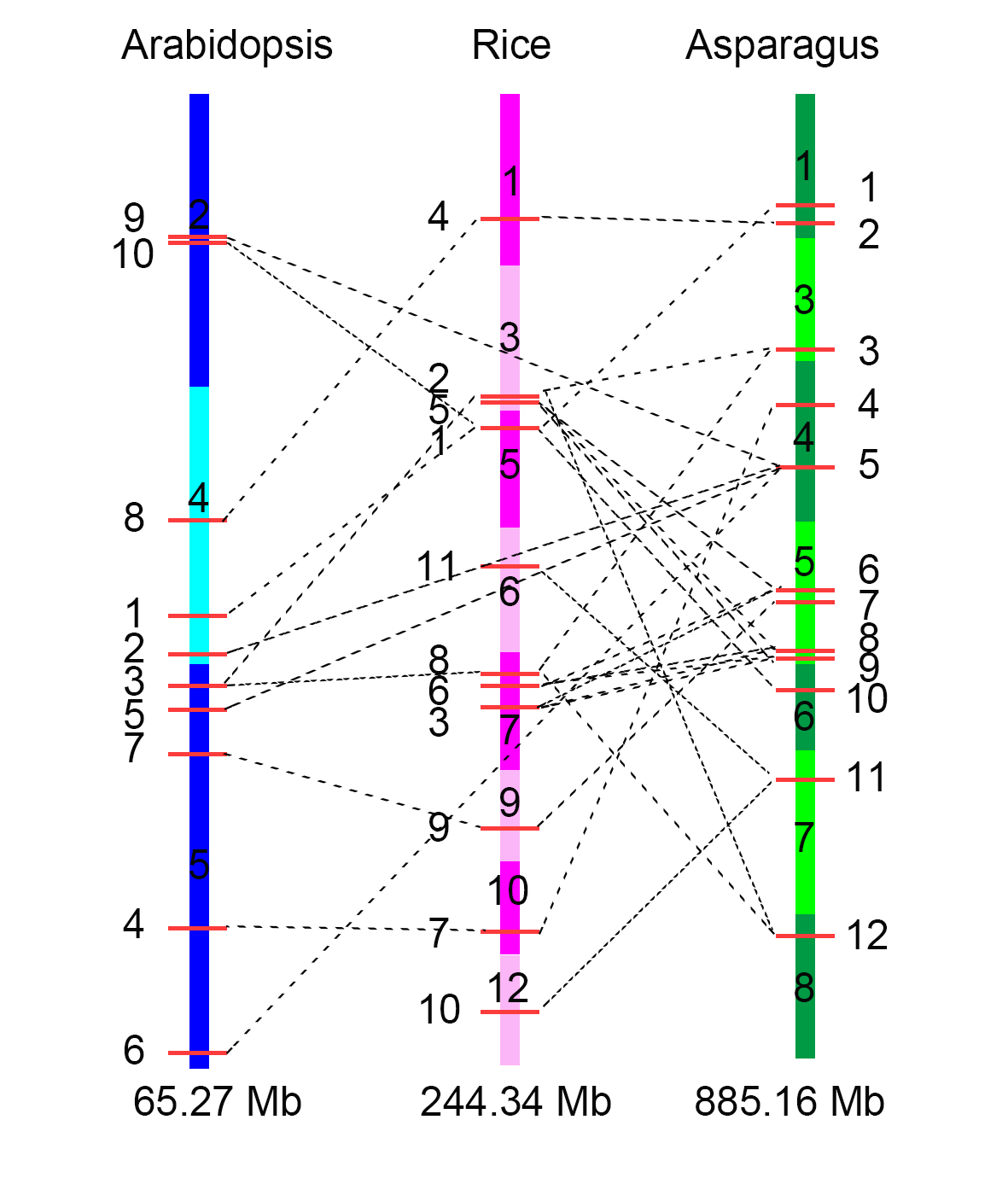

Supplement: Supplementary file 1 [file genes-10-00103-s001.zip › Figure S5.jpg]
